# The school-subject-specificity hypothesis: Implication in the relationship with grades

**DOI:** 10.1371/journal.pone.0230103

**Published:** 2020-04-21

**Authors:** Julien Chanal, Delphine Paumier

**Affiliations:** 1 Faculty of Psychology and Educational Sciences, University of Geneva, Geneva, Switzerland; 2 Distance Learning University, Brig, Switzerland; University of New England, AUSTRALIA

## Abstract

The aim of the present study was to examine the implication of the differences in autonomous and controlled motivation specificity in their relationships with student’s grades. The school-subject-specificity hypothesis postulates that the more autonomous the regulation is, the more specific to a school subject it is. 579 junior high school children were asked to complete a questionnaire assessing their motivation at the academic level as well as at the situational level (i.e., French, mathematics, English, and physical education), both simultaneously. As expected, results from structural equation modeling revealed that autonomous motivation was more specific to the situational level than controlled motivation. Moreover, results showed that the more specific the regulations are, the more relationships with students’ grades can be found. Therefore, this study offers a new understanding of previous results between autonomous and controlled regulations with grades and of the relationships between academic self-concepts, academic achievement and motivation.

## Introduction

Self-determination theory (SDT) endorses an organismic viewpoint that describes two categories of motivation that can drive behaviors at school: autonomous and controlled. Autonomous motivation refers to behaviors that are experienced as volitional, choiceful, and endorsed at a high level of reflection whereas controlled motivation refers to behaviors carried out under internal or external pressure. During the past 40 years, an increasing number of studies using SDT show that when students are motivated by autonomous rather than controlled motivation, they experience more positive affective and learning outcomes (e.g., [1–3]). However, only a few studies have investigated various school subjects at the same time. Recently, it has been shown that autonomous and controlled motivation differed in their relative specificity to the situational level [4]. The present study was designed to explore more deeply the relations between autonomous or controlled motivation and student’s grades relatively to the school-subject-specificity hypothesis [4]. Therefore, in this study, we considered secondary students’ grades and autonomous and controlled motivation at a contextual level (i.e., in school in general) as well as in different school subjects (i.e., French, mathematics, English and physical education) simultaneously.

### Autonomous and controlled motivation

SDT postulates that there are different reasons for students to engage in school activities (e.g.,[2]). These reasons vary in terms of level of self-determination (i.e., the degree to which the regulation is integrated into the self). Intrinsic motivation occurs when students engage in a given activity because it is naturally satisfying in itself. Extrinsic motivation occurs when students are motivated by the external consequences of being engaged in a given activity (e.g.,[2]). SDT suggests that different regulation types of extrinsic motivation exist. From high to low self-determination (hereafter the motivational continuum), these types are identified, introjected, and external regulations. One of the most self-determined forms of extrinsic motivation is identified regulation, whereby individuals engage in a behavior because of the inherent value they attach to it: they perform behaviors by choice or because they consider them important. This is followed by introjected regulation, which refers to behaviors intended either to act self-protectively and to project a positive image to others or to avoid guilt and shame. Finally, external regulation refers to behaviors enacted under external sources of control such as pursuing rewards or avoiding punishments and constraints. As conceptualized in recent studies [5,6], an approach (act self-protectively or pursuing rewards) or an avoidance (avoid guilt or avoid punishments) orientation of introjected and external regulations could drive behavior and lead to different outcomes. Autonomous motivation encompasses intrinsic motivation and identified regulation, whereas controlled motivation includes introjected and external regulations.

### Between-school-subjects and hierarchical aspects of autonomous and controlled motivation

To date, only two studies [4,7] have examined autonomous and controlled motivation in different school subjects simultaneously. In their study, Guay et al. [7] investigated variations in motivation toward specific school subjects (i.e., between-school-subjects differentiation). They show that correlations between regulation types in different school subjects were different depending on the position of the regulation on the motivational continuum. Results show that between-school-subjects differentiation (i.e., the size of the correlations connecting regulations between school subjects) was stronger for autonomous than for controlled motivation. Specifically, correlations among intrinsic motivations towards three school subjects (mathematics, reading, and writing) were lower than correlations among the identified regulations for these school subjects. Moreover, correlations among identified regulations for these school subjects were lower than correlations among controlled motivations (introjected and external regulation were considered together).

Chanal and Guay [4] explored this differentiation effect considering the hierarchical aspect of motivation in school. Within the SDT framework, the Hierarchical Model of Intrinsic and Extrinsic Motivation (HMIEM) [8] postulated that motivation exists at three different hierarchical levels. These levels are described as: the *global*, the *contextual* and the *situational*. The global motivation level refers to the general orientation to engage in an activity according to an intrinsic, extrinsic or amotivated way [8]. The contextual motivation level refers to the tendency to be motivated toward a specific sphere like the academic domain [8]. The situational motivation level refers to the motivation when people practice a particular activity of this domain [8]. Considering these different levels at the same time, Chanal and Guay [4] proposed the *school-subject-specificity hypothesis* to explain why autonomous motivation is more differentiated than controlled motivation between school subjects. This hypothesis postulates that autonomous motivation is more differentiated than controlled motivation because it is more related to the situational level (i.e., more school-subject-specific) than controlled motivation. In two studies, they tested the school-subject-specificity hypothesis in four situational school subjects (mathematics, French, English and physical education) and at the contextual level (school). Results of the two studies supported this hypothesis. Firstly, they found that autonomous motivation measures were more specific to the situational school subject level than were the controlled motivation measures. They also found that they were less related to the contextual level than were the controlled motivation ones. Moreover, they found that correlations between differentiated-known constructs (i.e., student’s self-concepts) were more related to autonomous than controlled motivation. Globally, these results demonstrated for the first time that regulation types behave differently in their specificity depending on their position on the motivational continuum.

### Autonomous and controlled motivation relationships with grades

Research using SDT perspective shows that students who are autonomously motivated experience more positive behavioral, affective and cognitive outcomes [1,2,9] in educational environments. However, studies investigating the relation between autonomous or controlled motivation and academic achievement brought mixed and inconsistent evidence depending on the operationalization of motivation used (i.e., regulation types considered separately vs. combined) and the hierarchical level considered (i.e., in situational vs. contextual level) in these studies.

In research considering either combinations of all regulation types into a single composite score, autonomous and controlled motivation separately, or with a person-centered approach, results are consistent whatever the hierarchical level considered is. In studies using a Relative Autonomy Index (RAI, a composite score considering all the regulation types), results demonstrated that the higher the score was, the higher achievement in school in general (e.g., [10,11]). Only one published study [12] also demonstrated this result at the school subject level (i.e., in organic chemistry). In studies considering autonomous and controlled motivation as two broad categories, results confirmed positive link between autonomous motivation and achievement and also demonstrated a negative relation between controlled motivation and achievement (e.g., [13]). In studies using a person-oriented approach [14,15 (studies 1 and 2), 16] results confirmed the positive effect of self-determined profiles on grades with a notable exception. In Ratelle et al. [15 (study 3)], they find one profile characterized by high levels of both controlled and autonomous motivation and another profile characterized by high level of autonomous motivation and low level of controlled motivation that were not distinguish in studies 1 and 2. When comparing these two groups of individuals, authors failed to find differences in academic achievement between them.

In research investigating the effects of each regulation type proposed by SDT on academic achievement separately, results are less consistent. Taylor et al. [3 (study 1)] recently conducted a meta-analysis on this issue. They demonstrated that intrinsic motivation and identified regulation were positively associated with students’ grades, whereas introjected regulation, external regulation and amotivation were negatively related when considering 18 studies. However, these differentiated links according to the regulation type were not confirmed in all studies at the school level (e.g., [17,18 (studies 2, 3 and 4)]) and contrasting evidence appeared in other studies when relations were considered at the school subject level (e.g., [4,19,20]). For example, Chanal and Guay [4 (study 1)] showed positive links between achievement and matching intrinsic motivation for mathematics and reading but not for science and writing. Moreover, no significant correlations were found between identified regulation and students’ achievement assessed by teachers. Finally, their results for introjected and external regulations suggested that the relation between regulation types and achievement depended on the hierarchical level they were assessed. Indeed, whereas introjected and external regulations were negatively related to achievement at the contextual level, no significant correlations were found between these regulations and students’ achievement at the situational level.

### The present study

The main goal of the present study was to examine the relation of autonomous or controlled motivation to students’ grades. As previously discussed, studies showed inconsistent results when regulation types were considered separately or combined and/or when multiple hierarchical levels were considered. Therefore, based on the school-subject-specificity hypothesis and on a statistical modeling permitting to disentangle variance at different levels of measurements, we hypothesized that the correlations between autonomous and controlled motivation with student’s grades will be dependent upon the specificity of the measures assessed. Therefore, we conducted a study among secondary students in which we measured autonomous and controlled motivation at the contextual level (i.e., school in general) and in four school subjects (i.e., French, mathematics, English, and physical education).

This main goal will first permit us to replicate results concerning the school-subject-specificity hypothesis proposed by Chanal and Guay [4]. Secondly, it will permit us to investigate the sources of variance of controlled motivation measures. Indeed, controlled motivation was described as more contextual by the authors because most of the shared variance of the measures was located at the contextual level. However, this conclusion could be fallacious because of the modeling used. Indeed, shared variance between similar items has not been considered in this previous study. This is problematic, because item-specific variance (i.e., variability in the subjects’ answers that is due to the particular item assessed) could have not been distinguished from variance shared at the contextual level. Because we were interested in disentangling these multiple sources of variance (i.e., situational, contextual and item) to better understand the specificity of controlled motivation and to investigate the relationships with students’ grades in different school subjects, we considered items from various questionnaires used in the academic research for controlled motivation (See Fig 2 for an example in one regulation type). Consequently, we tested the school-subject-specificity hypothesis on a larger motivational continuum than Chanal and Guay (7 vs. 4 motivational regulations) and we also evaluated the correlations between students’ motivation assessments and students’ grades.

Our main hypothesis was that depending on their position on the motivational continuum, the regulations would be more or less specific to the situational level. That is, we were expecting autonomous motivation items to be more specific to the situational level than controlled regulations. This assumption was tested using the CTCM-1 model, in which the variance partitioning was decomposed between the contextual, the situational and the item levels.

## Method

### Participants and procedure

Participants were 579 French-Speaking students (50.1% male; mean age = 13.2 years, SD = 0.99 years) from a public junior high-school located in the Canton of Geneva, Switzerland. Participants were recruited between October and November 2013, and the study took place in December 2013 during teaching period.

An experienced research assistant administered questionnaires in the classroom with the following instructions given to all children: “This is a chance to help me find out how you feel. It is not a test. There are no right or wrong answers, and everyone will have different answers. I will ask you to read each question and then ask you to write down how you feel about it by circling a number on the scale ranging from 1 (*Totally disagree*) to 7 (*Totally agree*). Make sure your answers show how you feel about yourself. We will not show your answers to anyone else. If you do not understand a sentence or a word in a sentence, please tell me.”

This study was approved by the "Commission de recherche dans les écoles (school research commission)" of canton of Geneva. Written consent form was required from parents and children in order to participate in the study. The data has been obtained and analyzed anonymously. When study was approved by the "Commission de recherche dans les écoles", schools where to conduct the study has been provided by the administration. Therefore, we were attributed the public junior high-school where the study was conducted and cannot consider that our sample was representative of a larger population.

### Measures

#### Academic motivation

The regulation types were assessed with a paper and pencil questionnaire recently developed by Chanal, Cheval, Courvoisier and Paumier [21]. This questionnaire consists of different items extracted from scales classically used in SDT research in the academic domain or in physical education classes: the Academic Motivation Scale (AMS), the Academic Self-Regulation Questionnaire (A-SRQ), the Behavioral Regulation Questionnaire (BREQ), the Exercise Motivation Scale (EMS) and the Sport Motivation Scale (SMS). Most of them were issued from the French version of the AMS [22] and from items used for introjected approach and avoidance by Assor et al. [5] and Gagné et al. [6]. We selected a minimum of four items for each regulation types based on the most representative ones found in the different scales presented above. The same items were used to assess each regulation at the school subject and contextual levels (mathematics, French, English and physical education). The final tool for each school subject contains 29 items (see Table 3) divided in seven subscales: intrinsic motivation to experience stimulation (e.g., “Because I am having fun in …”; αs between .80 and .93), intrinsic motivation for achievement (i.e., a combination of intrinsic motivation to know and intrinsic motivation toward accomplishments) (e.g., “Because I feel pleasure when I progress in …”; αs between .82 and .91), identified regulation (e.g., “Because I consider that … is important”; αs between .79 and .86), introjected approach regulation (e.g., “Because I want to be satisfied with myself”; αs between .72 and .82), introjected avoidance regulation (e.g., “Because I would feel guilty if I did not do everything that I could”; αs between .66 and .75), external approach regulation (e.g., “To get good grades”; αs between .70 and .78) and external avoidance regulation (e.g., “To avoid bad grades”; αs between .71 and .74). Students were asked to rate how much they agreed with each item on a seven-point scale from 1 (*Totally disagree*) to 7 (*Totally agree*).

#### Students’ grades

School administration was asked to provide us with the students’ grades at the end of the year in each of the four school subjects as well as a mean of grades in all the school subjects. The use of standardized tests would have been a more reliable measure, however, we did not have access to standardized tests for all the school subjects assessed.

### Statistical analyses

#### Correlated trait-correlated method minus one model

The above-mentioned hierarchical and multidimensional structure of autonomous and controlled motivation requires statistical procedures that are able to handle these aspects simultaneously. As described in Chanal and Guay [4], the correlated trait-correlated method minus one (CTCM-1) model [23] appeared to be the most suitable modeling procedure for our research purpose. This model allows testing of the hierarchical structure of autonomous and controlled academic motivations while taking into account multiple school subjects. The CTCM-1 permitted us to disentangle multiple sources of variance in autonomous and controlled motivations in various school subjects (e.g., mathematics, French, English, and physical education). More specifically, autonomous and controlled motivations at the school level are considered as being a single trait, whereas motivations for various school subjects are considered as school subject deviations from the global trait. Each indicator for the four school subjects are therefore explained by specific latent constructs but also by a latent construct for contextual autonomous or controlled motivation. Consequently, the latent factor at the contextual level encompasses the common variance of the school motivation measures in the school subject indicators and the latent factors at the school subject level are specific deviations from the global trait. Crucially, the method factor is missing for indicators assessing each regulation at the contextual level, and thus allows the model to be identified and a to obtain a unique solution for all model parameters.

Factorial scores for each construct was then extracted and correlated with student’s grades.

#### Missing data

Less than 1% of the data were missing. We performed a full information maximum likelihood (FIML) estimation using Mplus (version 7).

#### Estimation and goodness of fit

All models were tested with maximum likelihood estimation using robust standard errors (MLR estimation). To determine the fit of the model, we used the comparative fit index (CFI), the Tucker-Lewis index (TLI), the root mean square error of approximation (RMSEA), and the standardized root mean square residual (SRMR). The CFI and TLI values close to or above 0.90 and RMSEA and SRMR values close to or below .08 are typically considered acceptable [24,25].

#### Parallel items

**Fig 1:**
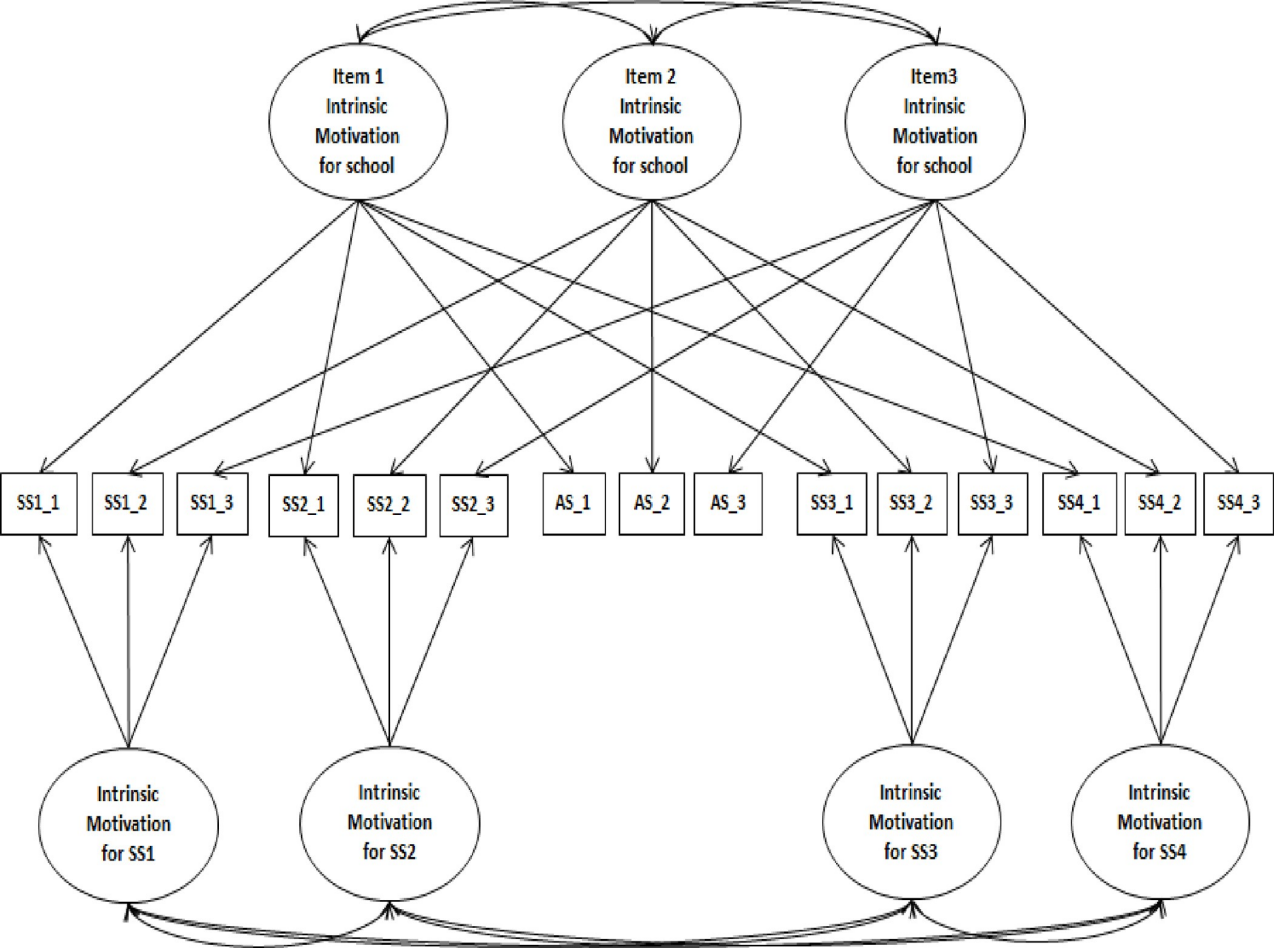
Correlated item-specific trait-correlated method minus one model for intrinsic motivation. SS1-SS4 = school subjects 1 to 4, SS1_1-SS1_3 = items for school subject 1, SS2_1-SS2_3 = items for school subject 2, AS_1-AS_3 = items for Academic, SS3_1-SS3_3 = items for school subject 3, SS4_1-SS4_3 = items for school subject 4.

**Fig 2:**
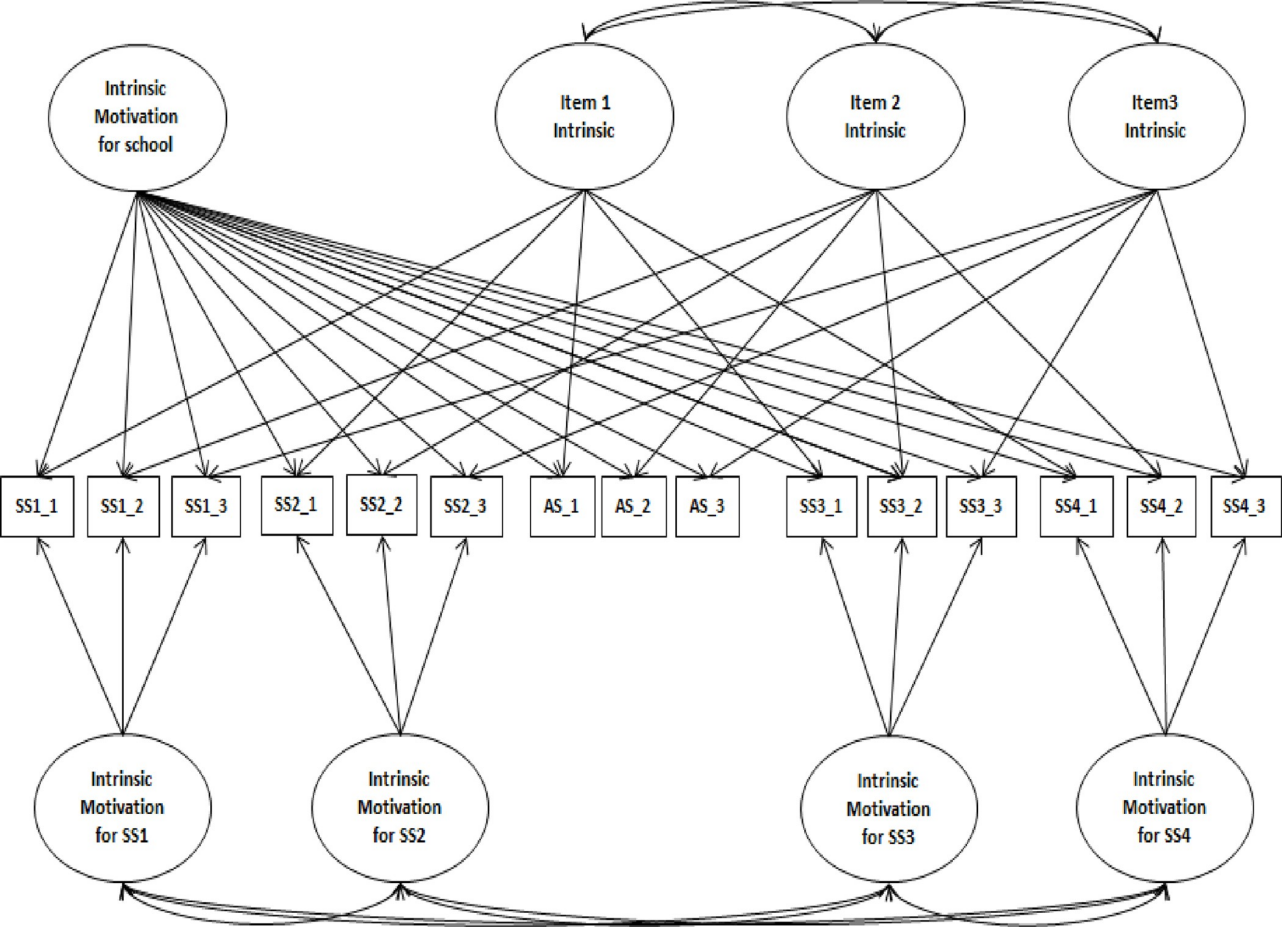
Correlated item-specific trait-correlated method minus one model for intrinsic motivation with item specific variance. SS1-SS4 = school subjects 1 to 4, SS1_1-SS1_3 = items for school subject 1, SS2_1-SS2_3 = items for school subject 2, AS_1-AS_3 = items for Academic, SS3_1-SS3_3 = items for school subject 3, SS4_1-SS4_3 = items for school subject 4.

Identical items were used to assess the same regulations across school subjects. Chanal and Guay [4] created an item-specific latent factor for the same item at the contextual level (see the example for intrinsic motivation in Fig 1). However, this modeling did not permit them to disentangle variance shared at the item level and at the contextual level. Therefore, we used a new modeling that considers variance shared at the item level and variance shared at the contextual level separately (see Fig 2).

## Results

Table 1 presents fit indices for each regulation type model (see Fig 2 for intrinsic motivation). All models show a good fit to the data.

**Table 1:** Fit indices of the models.

Regulation type	χ^2^	CFI	TLI	RMSEA	SRMR
Intrinsic motivation-stimulation	164.31	0.99	0.99	0.02	0.02
Intrinsic motivation-achievement	248.06	0.99	0.98	0.03	0.03
Identified	230.25	0.98	0.97	0.04	0.03
Introjected Approach	203.00	0.99	0.98	0.03	0.03
Introjected Avoidance	146.33	1.00	0.99	0.02	0.03
External Approach	353.31	0.98	0.97	0.03	0.03
External Avoidance	163.67	0.99	0.99	0.02	0.02

### The school-subject-specificity hypothesis

Table 2 presents the percentage of total variance of items that are attributed to the three levels considered as well as the residual variance.

**Table 2:** Percentage of the variance due to situational, contextual and item levels for each school subject and on average.

**Intrinsic-Stimulation**	**Situational**	**Contextual**	**Item**	**Residual**
**French**	40	6	31	22
**Mathematics**	52	13	16	20
**English**	58	9	11	21
**Physical education**	73	2	5	20
**Average**	56	8	16	21
**Intrinsic-Achievement**	**Situational**	**Contextual**	**Item**	**Residual**
**French**	23	28	29	21
**Mathematics**	27	34	16	27
**English**	47	19	9	25
**Physical education**	56	8	4	32
**Average**	38	22	15	26
**Identified**	**Situational**	**Contextual**	**Item**	**Residual**
**French**	30	24	15	31
**Mathematics**	42	22	14	23
**English**	43	16	9	32
**Physical education**	54	8	3	35
**Average**	42	18	10	30
**Introjected Approach**	**Situational**	**Contextual**	**Item**	**Residual**
**French**	11	35	26	28
**Mathematics**	16	33	27	23
**English**	24	25	23	28
**Physical education**	39	15	12	34
**Average**	23	27	22	28
**Introjected Avoidance**	**Situational**	**Contextual**	**Item**	**Residual**
**French**	9	30	33	28
**Mathematics**	14	26	36	24
**English**	19	25	28	28
**Physical education**	23	21	23	33
**Average**	16	26	30	28
**External Approach**	**Situational**	**Contextual**	**Item**	**Residual**
**French**	7	7	65	21
**Mathematics**	9	8	55	28
**English**	10	15	51	25
**Physical education**	17	19	30	34
**Average**	11	12	50	27
**External Avoidance**	**Situational**	**Contextual**	**Item**	**Residual**
**French**	5	20	53	22
**Mathematics**	7	22	44	27
**English**	9	16	55	20
**Physical education**	20	14	28	37
**Average**	10	18	45	27

Results confirm our hypothesis. Autonomous motivation appears to be more specific (between 38 to 56% in total variance) than controlled motivation (between 10 to 23% in total) in average (Table 2). Moreover, results demonstrated an almost perfect decrease pattern along the motivational continuum in situational variance except between intrinsic-achievement and identified regulation.

### The contextual and items level shared variance

The new modeling used in this study allowed us to disentangle contextual and item level shared variance. Analyses indicated (1) if regulation types were more contextual along the motivational continuum, (2) and if item-specific shared variance was similar along the motivational continuum. Results show that contextual variance does not increase along the motivational continuum continuously (Table 2). If on average, contextual shared variance seemed to increase from intrinsic motivation to introjected approach and avoidance (from 8% to 27%), it decreased with external approach and avoidance (12% and 18%). As residual variance was quite similar along regulation types (between 26 to 30% except for intrinsic-stimulation with 21%), items level shared variance is found to be responsible for this result. Indeed, if autonomous motivation shares only between 10 to 16% of variance at the item level, for controlled motivation, percentage variance at the item level fluctuates between 22 to 50%. Moreover, looking more finely at these results, it appears that variance shared at the item level tends to increase constantly along the motivational continuum (except for identified regulation).

### The relations between situational and items levels constructs and students’ grades

#### Situational correlations

Results about situational level constructs are presented in Table 3. Pearson correlations between each regulation type and grades are estimated in each school subject. For intrinsic motivation-stimulation and identified regulation, results show that correlations are significant for each of the four school subjects. Intrinsic motivation-achievement appears to have significant relationships for three school subjects. In total, on the 12 correlations between autonomous motivation and school grades, 11 (92%) are significant. For controlled motivation, on the 16 correlations between controlled motivation and students’ grades, only 8 (50%) are significant, and only 2 are in line with SDT theoretical predictions.

**Table 3:** Correlations between situation regulations and grades.

**Intrinsic-Stimulation**	**Grades**			
****	**French**	**Maths**	**English**	**Phys Ed**
**French**	.105*	*-*.150*	.*004*	*-*.*007*
**Mathematics**	*-*.*080*	.277*	*-*.196*	.121*
**English**	*-*.*049*	*-*.163*	.179*	*-*.*016*
**Physical education**	*-*.107*	*-*.*038*	*-*.139*	.366*
**Average**	Mean of convergent *r*s (diagonal *r*s)		.232	
	Mean of divergent *r*s (others *r*s)		-.035	
**Intrinsic-Achievement**	**Grades**			
	**French**	**Maths**	**English**	**Phys Ed**
**French**	.046	*-*.095*	*-*.*039*	*-*.*014*
**Mathematics**	*-*.*020*	.217*	*-*.091*	.125*
**English**	*-*.*012*	*-*.125*	.146*	.*003*
**Physical education**	*-*.*032*	.*021*	*-*.*082*	.307*
**Average**	Mean of convergent *r*s (diagonal *r*s)		.179	
	Mean of divergent *r*s (others *r*s)		-.004	
**Identified**	**Grades**			
	**French**	**Maths**	**English**	**Phys Ed**
**French**	.186*	.*061*	.093*	.*016*
**Mathematics**	.*054*	.219*	*-*.*055*	.*068*
**English**	.085*	*-*.*069*	.207*	.*032*
**Physical education**	*-*.*062*	*-*.*015*	-.081	.298*
**Average**	Mean of convergent *r*s (diagonal *r*s)		.228	
	Mean of divergent *r*s (others *r*s)		.033	
**Introjected Approach**	**Grades**			
	**French**	**Maths**	**English**	**Phys Ed**
**French**	.051	*-*.106*	.*013*	.*002*
**Mathematics**	.*003*	.114*	*-*.*024*	.*023*
**English**	*-*.*023*	*-*.153*	.128*	-.*006*
**Physical education**	*-*.*046*	*-*.*028*	-.*076*	.235*
**Average**	Mean of convergent *r*s (diagonal *r*s)		.132	
	Mean of divergent *r*s (others *r*s)		-.014	
**Introjected Avoidance**	**Grades**			
	**French**	**Maths**	**English**	**Phys Ed**
**French**	.029	*-*.*023*	.*052*	.*042*
**Mathematics**	.*042*	.019	.*065*	.*048*
**English**	.*002*	*-*.*051*	.127*	.*003*
**Physical education**	.*007*	.*023*	-.020	.219*
**Average**	Mean of convergent *r*s (diagonal *r*s)		.098	
	Mean of divergent *r*s (others *r*s)		.031	
**External Approach**	**Grades**			
	**French**	**Maths**	**English**	**Phys Ed**
**French**	-.018	*-*.154*	*-*.*062*	*-*.*006*
**Mathematics**	*-*.*067*	-.022	*-*.100*	*-*.*016*
**English**	*-*.*075*	*-*.156*	.028	*-*.*022*
**Physical education**	*-*.*078*	*-*.111*	*-*.100*	.204*
**Average**	Mean of convergent *r*s (diagonal *r*s)		.048	
	Mean of divergent *r*s (others *r*s)		-.057	
**External Avoidance**	**Grades**			
	**French**	**Maths**	**English**	**Phys Ed**
**French**	-.081	*-*.*060*	*-*.*043*	*-*.*079*
**Mathematics**	*-*.161*	-.113*	*-*.112*	*-*.*069*
**English**	*-*.125*	*-*.*054*	-.140*	*-*.*030*
**Physical education**	*-*.089*	*-*.*022*	*-*.*072*	-.028
**Average**	Mean of convergent *r*s (diagonal *r*s)		-.091	
	Mean of divergent *r*s (others *r*s)		-.073	

#### Item correlations

Results about items correlations with student’s grade are presented in Table 4. Pearson correlations between each item of the motivation questionnaire and grades in different school subjects were estimated. In total, out of the 42 correlations between items and grades for autonomous motivation, only 7 (17%) are significant. More specifically, 5 of the 7 correlations between grades and autonomous motivation are found in French, which is the less specific school subject (see Table 2). These are all in line with SDT predictions except for one item of identified regulation (“Because I consider it important to feel good”) that is negatively related to grades in mathematics, French and English.

**Table 4:** Correlations between grades and regulations at the item-specific level. Only significant correlations are reported.

	Grades			
	French	Maths	English	Phys Ed
Because … brings me pleasure	.11			
Because I am having fun in …	.10			
Because it is nice for me to learn in …				
Because I like this	.09			
Because I feel pleasure when I progress in …				
Because I discover new things …				
Because I have pleasure in feeling more competent in …	.09			
Because I find new interesting elements to learn in …				
Because I consider that … is important				
Because I consider it important for later				
Because I find it important to me				
Because I consider it important to feel good	-.10	-.09	-.15	
In order to feel proud of myself				
Because I want to be satisfied with myself				
In order to impress others	-.18	-.11	-.15	
In order to feel good with myself				.09
Because otherwise I will be ashamed of myself				
Because I would feel guilty if I did not do everything that I could				.09
Because I do not want to disappoint				
Because I do not want to be rejected	-.17	-.08	-.16	
To get good grades	.14		.12	.08
To please other people	-.14		-.09	
So that other people appreciate me	-.21	-.13	-.15	
So that you think I am good at …				
To get rewards	-.09			
To avoid bad grades	.15		.13	.10
To avoid being punished	-.10		-.10	
To avoid trouble	-.19	-.13	-.15	
To avoid being reproached		-.09		

For controlled motivation, out of the 68 correlations between items and grades, 26 (38%) are significant. More specifically, 8 of the 26 are found for introjected approach and avoidance and 18 of the 26 for the external approach and avoidance regulation types, which are the less specific regulation types. Twelve items of controlled motivation are correlated to students’ grades. Eight are negatively correlated, and 4 are positively correlated. Notably, two of these items, explicitly referring to grades (“To get good grades” and “To avoid bad grades”) are each positively related to students’ grades in three different school subjects (French, English and physical education).

## Discussion

The aim of this study was to examine the implication of the school-subject-specificity hypothesis [4] for the relationships between autonomous and controlled motivation and student’s grades.

### The school-subject-specificity hypothesis

Results confirm the school-subject-specificity hypothesis. Autonomous motivation is more school-subject-specific than controlled motivation, replicating Chanal and Guay [4] results. Our study obtains additional evidence of this effect with more regulation types (7 against 4). As expected, results demonstrate that specificity of the regulation types increases along the motivational continuum. However, contrary to Chanal and Guay [4], our results show that controlled motivation is not more related to the contextual level than autonomous motivation. Indeed, our study show that variance in the assessments of some regulation types of controlled motivation is more importantly related to items level than to contextual or situational levels. These results have serious implications concerning the SDT researches in the academic domain. First, they highlight the necessity to consider simultaneously motivation for different school subjects independently at the same time as well as contextual motivation to fully capture the interrelation between autonomous and controlled motivation for different school subjects. Second, they questioned the use of composite scores in determining subjects’ situational motivation in a particular school subject. Indeed, creation of composite scores combining together autonomous and controlled motivation that are not equally specific to situational level will predominantly express variations of autonomous motivation between subjects in this situational school subject. Therefore, results could either conduct to attribute between subjects’ differences on global self-determination instead of autonomous motivation only, or reduce between subjects’ variability in autonomous motivation and contribute to type II errors. For us, these results could help to explain inconsistencies found in the academic literature.

### On the relation of autonomous and controlled motivation to grades

Our results allow us to suggest that the relations between academic motivation and grades are dependent (1) on the specificity of the constructs and (2) of the items used in the questionnaires. Indeed, correlations between autonomous and controlled motivation at the situational level are dependent on the specificity of the construct. The more specific the school subject measures are, the more relations with grades are found. However, these relations are not fully in line with classical SDT perspective. Indeed, if we consistently find positive links between autonomous motivation and grades (i.e., for more specific constructs) at the situational level, results are mixed for controlled motivation. Among the 8 correlations found to be significant, 6 are positive and 2 are negative. These results echo with results obtained at the items level. Indeed, among the 12 items measuring autonomous motivation, only 5 are related to grades. Among these 5, 4 are positively related to grades in line with SDT perspective, and only 1 is negatively related. Therefore, for autonomous motivation, there is a general accordance between results at the situational and items levels, with more relations at the school subject level because autonomous motivation is globally more specific. In the controlled motivation side, among the 17 items measuring it, 12 items are related to grades. Among these 12, 8 are negatively related to grade in line with SDT perspective, and 4 are positively related. Therefore, for controlled motivation, there is no general accordance between results at the situational and items levels. At the school subject level, we find more positive correlations than negative ones whereas at the items level, we find more negative correlations that positive ones. However, if we consider items that do not strictly refer to grades at the item level, only negative correlations are found between controlled motivation and grades at the items level. For us, these results show that mixed evidence found in the literature concerning relation between controlled motivation and grades at the situational level could be due to the different items used in various questionnaires (e.g., AMS vs. A-SRQ), in addition to the fact that controlled motivation is not specific to the situational level.

### Implications of these results

The multidimensionality and hierarchical aspects of student’s academic self-concept investigated over the past 30 years (e.g., [26]), implied a lot of internal and external comparison processes interfering with and causing academic achievement (see [26] for a review on The Reciprocal Effects Model (REM), and the Frame of Reference Models). For SDT researchers, this new area of research appears to be far more complex because of the motivational continuum and the multiple regulation types described. Indeed, as autonomous and controlled motivation are not equally specific to the hierarchical level (or dimension in Marsh’ terminology) in which they are assessed, this implies that some regulations would be involved in reciprocal effects model and/or frame of reference models at the situational level (i.e., the autonomous ones) whereas others will or will not (i.e., the controlled ones) depending on their level of specificity.

Taylor et al. [3] results give us some first evidence of the implication of the specificity hypothesis on the REM between motivation and academic achievement. Indeed, its results show that intrinsic motivation was the only regulation type in the three studies that was related to achievement at Time 2 controlling for baseline achievement. Moreover, in Study 2, achievement at Time 1 also predicted intrinsic motivation at Time 2 controlling for previous intrinsic motivation, demonstrating complete REM between intrinsic motivation and achievement. This result is not surprising considering that intrinsic motivation is the most specific motivation and therefore could more easily interfere and cause academic achievement. However, these studies did not consider students’ self-concept.

One previous study already attempted to consider student’s self-concept, motivation and achievement to determinate the causal sequence between these [27]. However, this study considered (1) only one dimension of motivation and (2) RAI composite score to operationalize motivation. Therefore, according to the between-school-subject hypothesis, and the differentiation in specificity for autonomous and controlled motivation, we consider that the conclusions drawn from this study should be considered cautiously.

Our results also have important implications for teachers’ practices. Indeed, because of the school-subject-specificity of the regulations, teachers’ autonomy-supportive behaviors in a school subject could affect only autonomous motivation but could have no impact on controlled motivation. Therefore, it is important to determine how teachers’ behaviors could be differently linked to student’s autonomous and controlled motivation regarding the school-subject-specificity results presented in this study.

### Limitations and future directions

This study has some limitations that must be considered when interpreting the findings. A first limitation is the cross-sectional design used in this study which do not permit us to infer about causality. Moreover, we also have to acknowledge that it was not possible to obtain standardized test for school subjects assessed and this also could have impact our results. In future research, it will be important to understand how antecedents and consequences at the situational level are impacted differently by autonomous and controlled motivation. Moreover, some additional research will be important to conduce in teacher formation interventions. Indeed, in line with our research, Guay et al. [28] showed that a teacher intervention program in the writing class was only linked to a student’s increase in intrinsic motivation. It would be then important to understand if specific interventions should be built to influence both students’ autonomous and controlled motivation.

## Conclusion

This study aimed to show an implication of a new hypothesis relative to the SDT perspective of student’s motivation in the academic domain. Results supported the school-subject-specificity hypothesis [4]. Moreover, this study extended previous research by devoting attention to the implications of this hypothesis to the question of the relation between autonomous and controlled motivation with academic achievement. Indeed, mixed results were found in the SDT literature concerning this particular outcome. Our results suggest that the specificity of the regulation types could be responsible for the relation found with students’ grades. The more specific the construct is, the more relations exist. Finally, our results demonstrate that relations between regulation types and outcomes in SDT could be due to the wording of the items more than the regulation per se. Indeed, we found in the same time positive and negative effect of introjected and external regulation items on students’ grades.

This study opens new research perspectives in our comprehension of the development of academic achievement across the educational background from a motivational perspective.

## Supporting information

S1 Dataset Data_Chanal_Paumier_2019. The dataset of the study.
(XLSX)
